# Differential Effects of Exogenous Glomalin-Related Soil Proteins on Plant Growth of Trifoliate Orange Through Regulating Auxin Changes

**DOI:** 10.3389/fpls.2021.745402

**Published:** 2021-09-20

**Authors:** Rui-Cheng Liu, Wei-Qin Gao, Anoop Kumar Srivastava, Ying-Ning Zou, Kamil Kuča, Abeer Hashem, Elsayed Fathi Abd_Allah, Qiang-Sheng Wu

**Affiliations:** ^1^College of Horticulture and Gardening, Yangtze University, Jingzhou, China; ^2^ICAR-Central Citrus Research Institute, Nagpur, India; ^3^Department of Chemistry, Faculty of Science, University of Hradec Kralove, Hradec Kralove, Czechia; ^4^Botany and Microbiology Department, College of Science, King Saud University, Riyadh, Saudi Arabia; ^5^Plant Production Department, College of Food and Agricultural Sciences, King Saud University, Riyadh, Saudi Arabia

**Keywords:** auxin, carrier protein, citrus, glomalin, mycorrhiza, IAA, transporter protein

## Abstract

Multiple functions of glomalin released by arbuscular mycorrhizal fungi are well-recognized, whereas the role of exogenous glomalins including easily extractable glomalin-related soil protein (EE-GRSP) and difficultly extractable glomalin-related soil protein (DE-GRSP) is unexplored for plant responses. Our study was carried out to assess the effects of exogenous EE-GRSP and DE-GRSP at varying strengths on plant growth and chlorophyll concentration of trifoliate orange (*Poncirus trifoliata*) seedlings, along with changes in root nutrient acquisition, auxin content, auxin-related enzyme and transporter protein gene expression, and element contents of purified GRSP. Sixteen weeks later, exogenous GRSP displayed differential effects on plant growth (height, stem diameter, leaf number, and biomass production): the increase by EE-GRSP and the decrease by DE-GRSP. The best positive effect on plant growth occurred at exogenous EE-GRSP at ½ strength. Similarly, the GRSP application also differently affected total chlorophyll content, root morphology (total length, surface area, and volume), and root N, P, and K content: positive effect by EE-GRSP and negative effect by DE-GRSP. Exogenous EE-GRSP accumulated more indoleacetic acid (IAA) in roots, which was associated with the upregulated expression of root auxin synthetic enzyme genes (*PtTAA1, PtYUC3*, and *PtYUC4*) and auxin influx transporter protein genes (*PtLAX1, PtLAX2*, and *PtLAX3*). On the other hand, exogenous DE-GRSP inhibited root IAA and indolebutyric acid (IBA) content, associated with the downregulated expression of root *PtTAA1, PtLAX1*, and *PtLAX3*. Root IAA positively correlated with root *PtTAA1, PtYUC3, PtYUC4, PtLAX1*, and *PtLAX3* expression. Purified EE-GRSP and DE-GRSP showed similar element composition but varied in part element (C, O, P, Ca, Cu, Mn, Zn, Fe, and Mo) concentration. It concluded that exogenous GRSP triggered differential effects on growth response, and the effect was associated with the element content of pure GRSP and the change in auxins and root morphology. EE-GRSP displays a promise as a plant growth biostimulant in citriculture.

## Introduction

Arbuscular mycorrhizal fungi (AMF) in the soil are extensively reported to colonize the roots of roughly 80% of terrestrial plants, forming arbuscular mycorrhizas (AMs). AMs help the host plant to absorb water and nutrients, whereas the host delivers the photoassimilates to the mycorrhizal fungi in the root for their growth (Huang et al., 2021a). Such mycorrhizal symbiosis represents important functions on promoting plant growth, improving stress resistance, enriching rhizospheric microbial diversity, and stabilizing ecosystems (Zhao et al., 2015; Wang et al., 2018). Spores and mycelium of AMF release an N-linked glycoprotein (glomalin) into the soil, popularly known as glomalin-related soil protein (GRSP) according to the Bradford assay (Rillig, 2004; Rillig and Mummey, 2010). GRSP is primarily divided into two fractions, viz., easily extractable GRSP (EE-GRSP) and difficultly extractable GRSP (DE-GRSP) (Koide and Peoples, 2013; Wu et al., 2014). EE-GRSP is considered to be newly produced by AMF and relatively more labile, whereas DE-GRSP originates from the turnover of EE-GRSP and thus represents a comparatively older glomalin of an inactive nature (Koide and Peoples, 2013; Wu et al., 2015a). Earlier studies indicated that GRSP promoted the storage of soil organic carbon (SOC), improved the distribution and stability of soil water-stable aggregate (WSA), enhanced the drought tolerance of plants, and reduced the metal toxicity of soil (Zou et al., 2014; Gao et al., 2019; He et al., 2020; Meng et al., 2020). The direct contribution of GRSP on WSA stability was much stronger than mycorrhizal extraradical hyphae or root mycorrhizal colonization (Rillig et al., 2001; Wu et al., 2014).

Studies have shown that GRSP was composed of 3–5% N, 36–59% C, 4–6% H, 33–49% O, 0.03–0.1% P, 0.8–8.8% Fe, and other cations (e.g., K, Ca, Si, Cu, and Mg), depending upon soil properties (Rillig et al., 2001; Lovelock et al., 2004; Nichols, 2008; Zhang et al., 2017a). Schindler et al. (2007) observed that the GRSP of peat soil contained aromatic hydrocarbons (42–49%), carboxyl groups (24–30%), and carbohydrates (4–16%) evident from the analysis of nuclear magnetic resonance (NMR). GRSP is, thus, a mixture, rich in various elements, essential to plant growth and development. Based on the mineral elements in GRSP, it is reasonable to hypothesize that GRSP as a biostimulant potentially improves plant growth responses. To confirm this hypothesis, Wang et al. (2015) firstly observed that the concentration of exogenous EE-GRSP was curvilinearly related with plant biomass yield in potted trifoliate orange (*Poncirus trifoliata* L. Raf.), with ½ strength of EE-GRSP exhibiting the highest magnitude of growth responses. Wu et al. (2015b) through a field study proved that exogenous EE-GRSP treatment produced a significant increase in SOC and soil phosphatase activity in 27-year-old Satsuma mandarin grafted on trifoliate orange. Chi et al. (2018) reported that ½ strength of exogenous EE-GRSP improved leaf gas exchange, iron-superoxide dismutase activity, abscisic acid, indole-acetic acid (IAA), and methyl jasmonate concentrations in leaves of potted trifoliate orange seedlings exposed to soil drought stress. These studies demonstrated the ability of exogenous EE-GRSP in improving plant growth response (Zou et al., 2015).

Although these experiments have shown an improved effect of exogenous EE-GRSP on plants, the underlying mechanisms remain still unclear. It is also unknown whether DE-GRSP, another fraction of GRSP, has a similar beneficial response on plant growth. Therefore, the present work aimed at evaluating the response of exogenous EE-GRSP and DE-GRSP at varying strengths on the growth of trifoliate orange seedlings, and at revealing the mechanism of GRSP affecting plant growth by analyzing the changes of auxins, gene expression, and mineral elements of purified GRSP fractions.

## Materials and Methods

### Preparation of Exogenous EE-GRSP and DE-GRSP

Soil samples were collected from a citrus orchard (located at the campus of Yangtze University), air-dried, sieved through 4-mm nylon, incubated with 20 mmol/L citrate buffer (pH 7.0) (m:v = 1:8) at 121°C and 0.11 MPa for 30 min and centrifuged at 10,000 × *g*/min for 5 min. The supernatant was collected as an exogenous full-strength EE-GRSP solution. The residues of EE-GRSP extraction in the centrifugal tube was continued to incubate with the same volume of 50 mmol/L sodium citrate buffer (pH 8.0) at 121°C and 0.11 MPa for 60 min, and centrifuged at 10,000 × *g*/min for 10 min (Wu et al., 2015a). The second supernatant was collected as a full-strength DE-GRSP solution. The soluble protein concentration was 0.24 and 0.36 mg/L in full-strength EE-GRSP and DE-GRSP solution, as put forward by the Bradford (1976) assay. A total of about 12 L of full-strength EE-GRSP and DE-GRSP solution were collected, respectively, along with 1.5 kg of the soil used.

### Experimental Design

In a completely randomized design, the experiment consisted of seven treatments: (i) control with 20 mmol/L citrate buffer (pH 7.0) (0 GRSP), (ii) quarter-strength EE-GRSP (¼ EE-GRSP), (iii) half-strength EE-GRSP (½ EE-GRSP), (iv) full-strength EE-GRSP (1 EE-GRSP), (v) quarter-strength DE-GRSP (¼ DE-GRSP), (vi) half-strength DE-GRSP (½ DE-GRSP), and (vii) full-strength DE-GRSP (1 DE-GRSP). The ½ and ¼ EE-GRSP was diluted with the full-strength EE-GRSP with 20 mmol/L citrate buffers (pH 7.0), and the ½ and ¼ DE-GRSP was prepared using the full-strength DE-GRSP by diluting with 50 mmol/L citrate buffers (pH 8.0). Each treatment was replicated four times, and each replicate consisted of two pots, resulting in a total of 56 pots (corresponding to 168 seedlings).

### Plant Setup

Three four-leaf-old trifoliate orange seedlings grown in autoclaved sand were transplanted into a plastic pot (2.1-L), in which 1.6 kg of autoclaved (121°C, 0.11 MPa, 2 h) soil sieved with 4-mm-size nylon was supplied. After 2 weeks of plant acclimatization, exogenous GRSP treatments were initiated. A 50 ml of exogenous EE-GRSP or DE-GRSP solution as per the proposed strength was applied into the corresponding pot at weekly intervals, for a total of 16 times. The experiment was carried out in an intelligent artificial climate chamber (AGC-P, Zhejiang QiuShi Artificial Environment Co., Ltd., Hangzhou, China) from March 22 to July 21, 2018, where the photon? ux density was 900 μmol/m^2^/s, day/night temperature 28/20°C, and relative air humidity 68% during the experiment.

### Determination of Plant Growth and Chlorophyll Concentrations

Plant height, stem diameter, and leaf number were recorded before harvest. The seedlings were divided into shoots and roots. The intact roots were scanned using the Epson Perfection V700 Photo Dual Lens System (J221A, Seiko Epson Corporation, Jakarta Selatan, Indonesia). The scanned root images were analyzed using the WinRHIZO software (Regent Instruments Inc., Montreal, QC, Canada) to obtain root morphological variables including total length, surface area, and volume. Subsequently, shoot and root biomass was weighed after drying at 75°C for 48 h. The total chlorophyll concentration was determined by the procedure suggested by Arnon (1949).

### Determination of Root Nutrient Acquisition

Root samples were digested in H_2_SO_4_ and H_2_O_2_. Subsequently, N content was determined by a chemical analyzer (Smartchem 200, Scientific Instruments Limited, Weston, FL, USA), and other mineral elements were assayed with the help of the ICP-Spectrometer (IRIS Advantage, Thermo Fisher Scientific Inc., Waltham, MA, USA).

### Determination of Root Auxins

Root IAA and indolebutyric acid (IBA) were extracted according to the method outlined by Dobrev and Kamínek (2002) with minor modification. The 0.2 g of root samples was ground in liquid nitrogen, extracted by adding 1 ml of pre-cooled extracting solution (methanol, distilled water, and formic acid, 15/4/1, v/v/v) for 12 h at 4°C, and centrifuged at 8,000 × *g*/min for 10 min. The centrifuged residue was re-extracted with 0.5 ml of the extracting solution for 2 h at 4°C. After centrifugation, two supernatants were combined, evaporated at 40°C under reduced pressure to near dryness (~0.5 ml), and decolorized three times with 0.5 ml of petroleum ether. After discarding the upper ether phase, the down phase was evaporated to the dryness at 40°C under reduced pressure, and 0.5-ml mobile phase (methanol and ddH_2_O in a ratio of 2:3) was added to dissolve. The content of IAA and IBA was determined with the help of high-performance liquid chromatography (HPLC; LC-100, Shanghai Wufeng Scientific Instrument Co., Ltd., Shanghai, China). The injection volume was 10 μl, the flow rate was 0.8 ml/min, the column temperature was 35°C, the sampling time was 40 min, and the detection wavelength was 254 nm.

### Expression Levels of Genes Associated With IAA

The extraction of total RNA from the roots and the reverse transcription of RNA were carried out according to the protocol previously described by Liu et al. (2018a). Four genes associated with IAA synthesis (*PtTAA1, PtTAR2, PtYUC3*, and *PtYUC4*) and four influx transporter protein genes of auxins (*PtAUX1, PtLAX1, PtLAX2*, and *PtLAX3*) were associated with the IAA synthesis and transport in trifoliate orange (Liu et al., 2018b) and selected according to the genome database of trifoliate orange (http://citrus.hzau.edu.cn/orange/index.php) (Huang et al., 2021b). The primers (Table 1) of these selected genes were designed with Primer 5. The β*-actin* was used as the housekeeping gene. The quantitative reverse transcription (qRT)-PCR was carried out using the conditions described by Liu et al. (2018a). The relative quantitative expression of genes was calculated by the 2^−ΔΔCT^ method as suggested by Livak and Schmittgen (2001).

**Table 1:** Specific primer sequences of genes used in this study for quantitative reverse transcription (qRT)-PCR.

**Genes**	**Accession number**	**Forward primer (5^′^ → 3^′^)**	**Reverse primer (5^′^ → 39k^′^)**
*PtTAA1*	Pt4g006620	TTTGAGGCGTTTTGGAGGAA	TTGTTGATTGCTTCAGCGAGTT
*PtTAR2*	Pt3g005090	CACACACGGCACACCCCTA	GCCTCCCACTCCCCAGATC
*PtYUC3*	Pt9g009520	CCTTCAGGTTTAGCCGTTGC	GGAAGTTTGGAAGTTGGCAGA
*PtYUC4*	Pt1g005780	GACCATCTGGGTTAGCCGTTT	GTATTTTGGGAAGTTTTCAGGGA
*PtAUX1*	Pt6g013420	CTTGACTCTGCCCTATTCATTCTC	TGGACCCAGTAACCCATCAAGC
*PtLAX1*	Pt4g022040	TTGGCGGACATGCAGTGAC	CAGCGGCAGCAGAAGGAAT
*PtLAX2*	Pt8g001790	TGTGGGAAGATGGGTAGGGAC	TAGTVATGCTCGCCCACCC
*PtLAX3*	Pt5g008650	ATCACTTTCGCTCCTGCTGC	CAAACCCAAATCCCACCACTA
*β-Actin*	Pt7g003560	CCGACCGTATGAGCAAGGAAA	TTCCTGTGGACAATGGATGGA

*Gene accession number is from the genome database of trifoliate orange*.

### Determination of Nutrient Elements in Purified EE-GRSP and DE-GRSP

We chose to purify the full-strength GRSP solution in order to study the difference in nutrient composition between exogenous EE-GRSP and DE-GRSP. The purification of EE-GRSP and DE-GRSP was performed according to the method described by He et al. (2020). The extracted full-strength GRSP solution was precipitated with HCl solution, dissolved with NaOH solution, dialyzated in a dialysis bag (MD 44-1000) with ddH_2_O, centrifuged at 5,000 × *g*/min for 5 min, and then freeze-dried. The nutrient elements, viz., C, H, O, S, and N contents of purified EE-GRSP and DE-GRSP, were analyzed by the Automatic Element Analyzer (Euro Vector EA3000, Shanghai Woolong Instrument Co., Ltd, Shanghai, China), whereas other elements were determined as per the ICP-Spectrometer (Optima 8000, PerkinElmer, Melville, NY, USA).

### Statistical Analysis

The generated data were statistically analyzed by ANOVA through the SAS software (v8.1; SAS Institute Inc., Cary, NC, USA). The significant difference between treatments was compared with Duncan's multiple range test at 0.05 levels. Pearson's correlation coefficient was calculated with the help of the SAS software.

## Results

### Changes in Growth Performance

**Figure 1:**
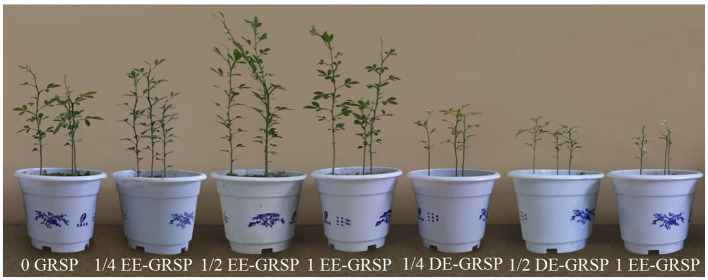
Growth performance of trifoliate orange by exogenous EE-GRSP and DE-GRSP at varying strength. DE-GRSP, difficultly extractable glomalin-related soil protein; EE-GRSP, easily extractable glomalin-related soil protein.

Compared with the control (0 GRSP), exogenous EE-GRSP significantly increased plant height, leaf number, stem diameter, and shoot and root biomass of trifoliate orange seedlings, with ½ EE-GRSP treatment having the most significant effect, increasing 17, 19, 22, 52, and 38%, respectively (Figure 1 and Table 2). By contrast, the growth response of DE-GRSP treatment was completely different from that of exogenous EE-GRSP, resulting in a significant inhibition in these plant growth parameters. The inhibiting effect of DE-GRSP on growth increased with the increase of DE-GRSP concentrations. Compared with 0 GRSP, 1 DE-GRSP significantly reduced plant height, leaf number, stem diameter, and shoot and root biomass by 61, 52, 50, 71, and 41%, respectively.

**Table 2:** Effects of exogenous GRSP on plant growth performance of potted trifoliate orange seedlings.

**Treatments**	**Plant height**	**Leaf number**	**Stem diameter**	**Biomass (g DW/plant)**	
	**(cm)**	**(#/plant)**	**(mm)**	**Shoot**	**Root**
0 GRSP	19.68 ± 0.22c	17.55 ± 0.19d	2.01 ± 0.08c	0.96 ± 0.07c	0.68 ± 0.04c
¼ EE-GRSP	21.83 ± 0.33b	19.13 ± 0.15b	2.59 ± 0.06a	1.02 ± 0.08c	0.73 ± 0.05b
½ EE-GRSP	23.06 ± 0.44a	20.85 ± 0.17a	2.45 ± 0.13a	1.46 ± 0.10a	0.94 ± 0.08a
1 EE-GRSP	21.47 ± 0.19b	18.58 ± 0.34c	2.22 ±± 0.14b	1.10 ± 0.11b	0.73 ± 0.07b
¼ DE-GRSP	10.63 ± 0.34d	9.93 ± 0.15e	1.51 ± 0.04e	0.37 ± 0.03d	0.53 ± 0.05d
½ DE-GRSP	8.83 ± 0.14e	9.00 ± 0.24f	1.77 ± 0.07d	0.32 ± 0.03d	0.49 ± 0.05d
1 DE-GRSP	7.67 ± 0.11f	8.50 ± 0.16f	1.00 ± 0.06f	0.28 ± 0.03e	0.40 ± 0.03e

*Data (mean ± SD, n = 4) followed by the different letters among treatments indicate significant differences at p < 0.05*.

*DE-GRSP, difficultly extractable glomalin-related soil protein; EE-GRSP, easily extractable glomalin-related soil protein*.

### Changes in Chlorophyll Concentration

**Figure 2:**
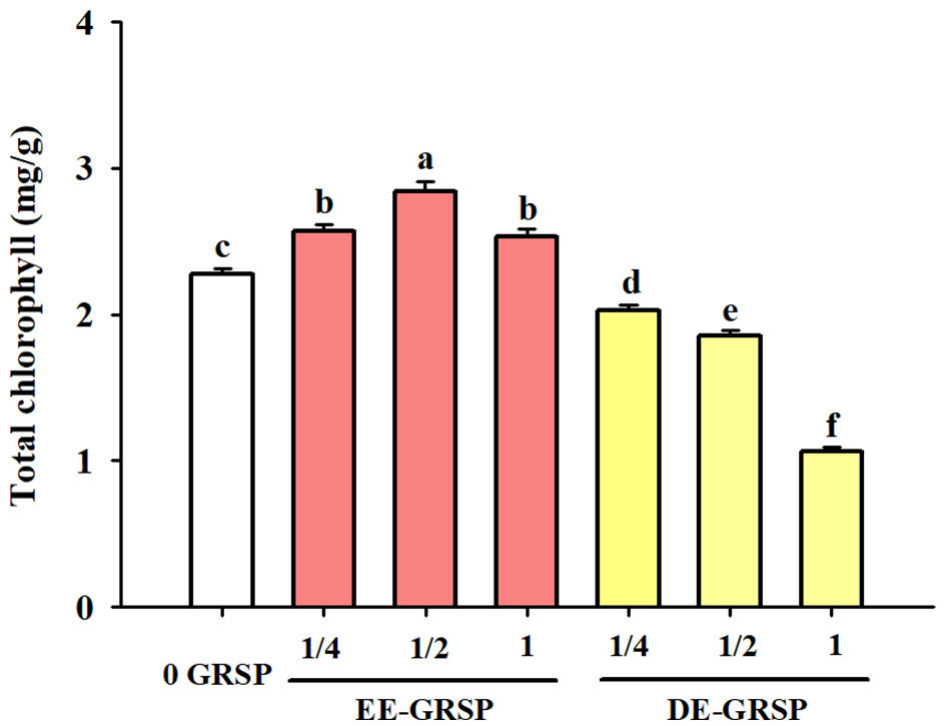
Effects of exogenous EE-GRSP and DE-GRSP on total chlorophyll concentration in leaves of trifoliate orange seedlings. Data (mean ± SD, *n* = 4) followed by different letters above the bars indicate significant differences (*p* < 0.05). DE-GRSP: difficultly extractable glomalin-related soil protein; EE-GRSP: easily extractable glomalin-related soil protein.

**Figure 3:**
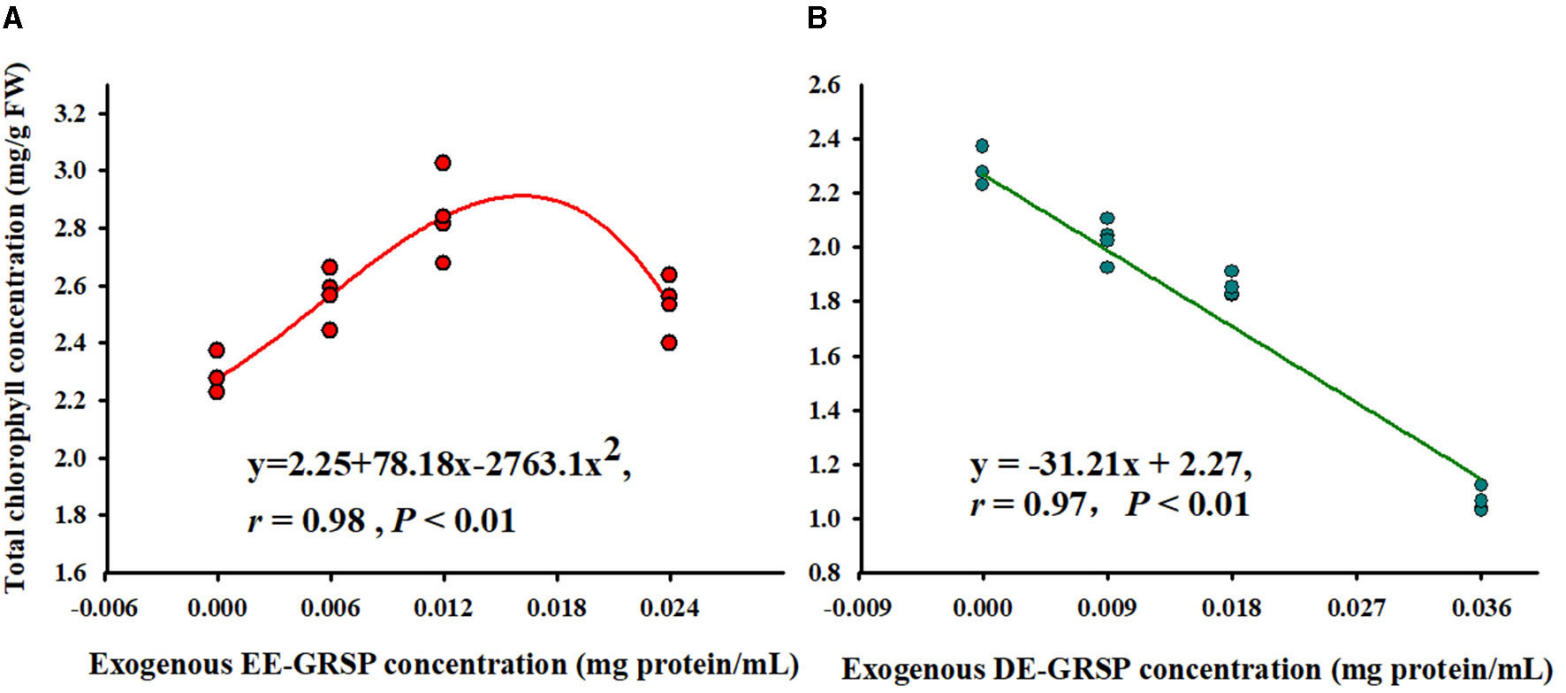
Correlation between exogenous EE-GRSP **(A)** and exogenous DE-GRSP **(B)** and total chlorophyll concentration (*n* = 16). DE-GRSP, difficultly extractable glomalin-related soil protein; EE-GRSP, easily extractable glomalin-related soil protein.

Application of exogenous EE-GRSP significantly increased leaf total chlorophyll concentration, independent of the concentrations, with ½ EE-GRSP treatment displaying the highest magnitude of response (Figure 2). Nevertheless, all the DE-GRSP treatments significantly reduced total chlorophyll concentration compared with 0 GRSP treatment. In a regression analysis between chlorophyll concentration and EE-GRSP and DE-GRSP concentration, a quadratic relationship was observed with exogenous EE-GRSP treatments, showing the highest total chlorophyll concentration occurring between 0.012 and 0.018 mg protein/ml (Figure 3A). And with exogenous DE-GRSP treatment conditions, total chlorophyll concentration was negatively and linearly correlated with DE-GRSP concentration (Figure 3B).

### Changes in Root Mineral Element Contents

Application of exogenous GRSP influenced the root nutrient acquisition to varying proportions. Compared with the control (0 GRSP), ¼ EE-GRSP treatment significantly increased root N, P, K, Ca, Mg, Zn, and Fe content by 7, 43, 42, 342, 8, 32, and 5%, respectively; ½ EE-GRSP treatment dramatically increased root N, P, K, Ca, Mg, Cu, and Fe content by 21, 23, 60, 314, 84, 50, and 12%, respectively; 1 EE-GRSP significantly increased root N, P, K, and Zn content by 12, 84, 12, and 35%, respectively (Table 3). However, compared with 0 GRSP, ¼ DE-GRSP treatment significantly reduced root N, P, K, Mg, Cu, Zn, and Fe content by 22, 31, 23, 17, 27, 22, and 78%, respectively; ½ DE-GRSP decreased root N, P, K, Mg, Cu, Zn, and Fe content by 21, 15, 34, 19, 3, 32, and 70%, respectively; and 1 DE-GRSP treatment reduced root N, P, K, Mg, Cu, Zn, and Fe content by 20, 29, 31, 25, 46, 32, and 59%, respectively. However, EE-GRSP and DE-GRSP displayed no significant effect on root Mn content.

**Table 3:** Effects of exogenous EE-GRSP and DE-GRSP on nutrient acquisition in roots of trifoliate orange seedlings.

**Treatments**	**N (%)**	**P (g/kg)**	**K (g/kg)**	**Ca (g/kg)**	**Mg (g/kg)**	**Cu (mg/kg)**	**Zn (mg/kg)**	**Mn (mg/kg)**	**Fe (mg/kg)**
0 GRSP	1.07 ± 0.02d	2.03 ± 0.02d	9.60 ± 0.42d	6.11 ± 0.25cd	3.09 ± 0.09c	14.14 ± 0.82b	11.68 ± 0.54b	47.68 ± 6.64a	1720.49 ± 31.61c
¼ EE-GRSP	1.14 ± 0.03c	2.90 ± 0.03b	13.66 ± 0.59b	27.03 ± 1.09a	3.35 ± 0.08b	16.35 ± 0.37b	15.38 ± 0.72a	46.73 ± 7.00a	1804.40 ± 32.99b
½ EE-GRSP	1.29 ± 0.03a	2.50 ± 0.03c	15.33 ± 0.66a	25.28 ± 1.04b	5.69 ± 0.14a	21.22 ± 5.67a	10.21 ± 0.48c	44.90 ± 6.73a	1927.29 ± 35.33a
1 EE-GRSP	1.20 ± 0.03b	3.73 ± 0.04a	10.76 ± 0.47c	6.20 ± 0.25cd	2.75 ± 0.07d	17.14 ± 3.31b	15.82 ± 0.74a	45.66 ± 6.83a	1752.08 ± 32.12c
¼ DE-GRSP	0.83 ± 0.01e	1.40 ± 0.02f	7.39 ± 0.32e	6.44 ± 0.26cd	2.56 ± 0.06e	10.27 ± 0.59c	9.07 ± 1.82c	46.80 ± 2.90a	380.40 ± 6.97f
½ DE-GRSP	0.85 ± 0.02e	1.72 ± 0.02e	6.38 ± 0.28f	7.02 ± 0.28c	2.49 ± 0.06e	9.95 ± 1.15c	7.99 ± 0.37d	42.78 ± 3.38a	520.82 ± 9.67e
1 DE-GRSP	0.86 ± 0.02e	1.44 ± 0.02f	6.59 ± 0.29f	5.83 ± 0.24d	2.32 ± 0.06f	7.63 ± 0.44d	7.97 ± 0.37d	44.25 ± 6.32a	703.47 ± 12.86d

*Data (mean ± SD, n = 4) followed by the different letters among treatments indicate significant differences at p < 0.05*.

*DE-GRSP, difficultly extractable glomalin-related soil protein; EE-GRSP, easily extractable glomalin-related soil protein*.

### Changes in Root Morphology

**Figure 4:**
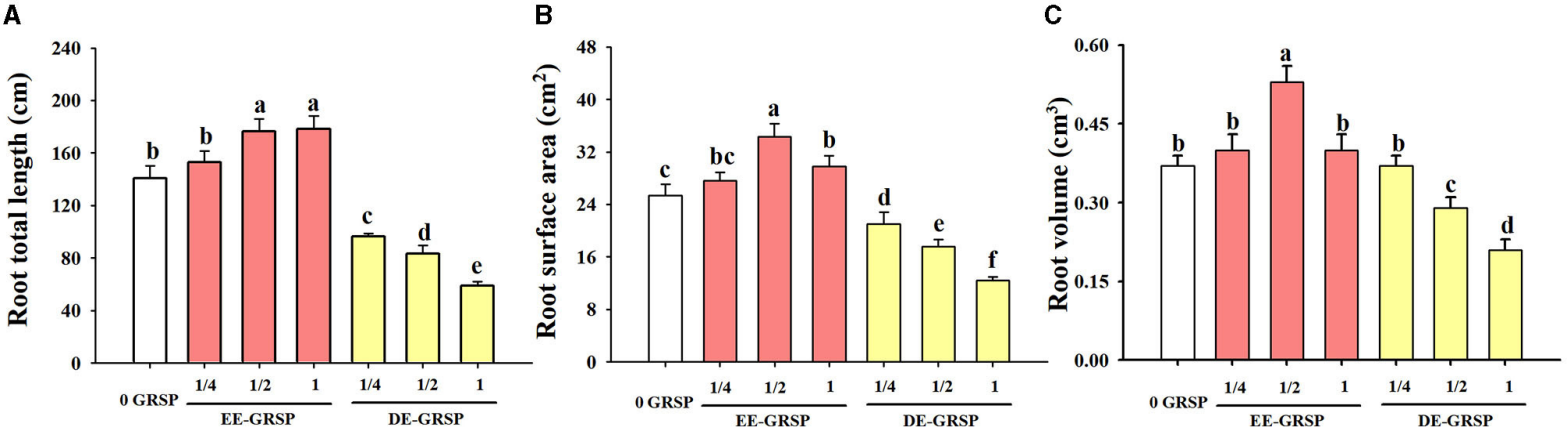
Effects of exogenous EE-GRSP and DE-GRSP on root total length **(A)**, root surface area **(B)**, and root volume **(C)** of trifoliate orange. Data (mean ± SD, *n* = 4) followed by different letters above the bars indicate significant differences (*p* < 0.05). DE-GRSP, difficultly extractable glomalin-related soil protein; EE-GRSP, easily extractable glomalin-related soil protein.

Compared with the control, ¼ EE-GRSP treatment had no significant effect on root total length, root surface area, and root volume, whereas ½ EE-GRSP treatment significantly increased root total length, surface area, and volume by 25, 35, and 43%, respectively (Figures 4A–C). Application of 1 EE-GRSP distinctly increased root total length and surface area by 27 and 17%, respectively, with no significant effect on root volume. Exogenous application of DE-GRSP, however, almost significantly decreased root total length, surface area, and volume compared to the control, with increasing DE-GRSP strength, except that there was no difference in root volume between ¼ DE-GRSP and the control.

### Changes in Element Composition of Purified GRSP

**Figure 5:**
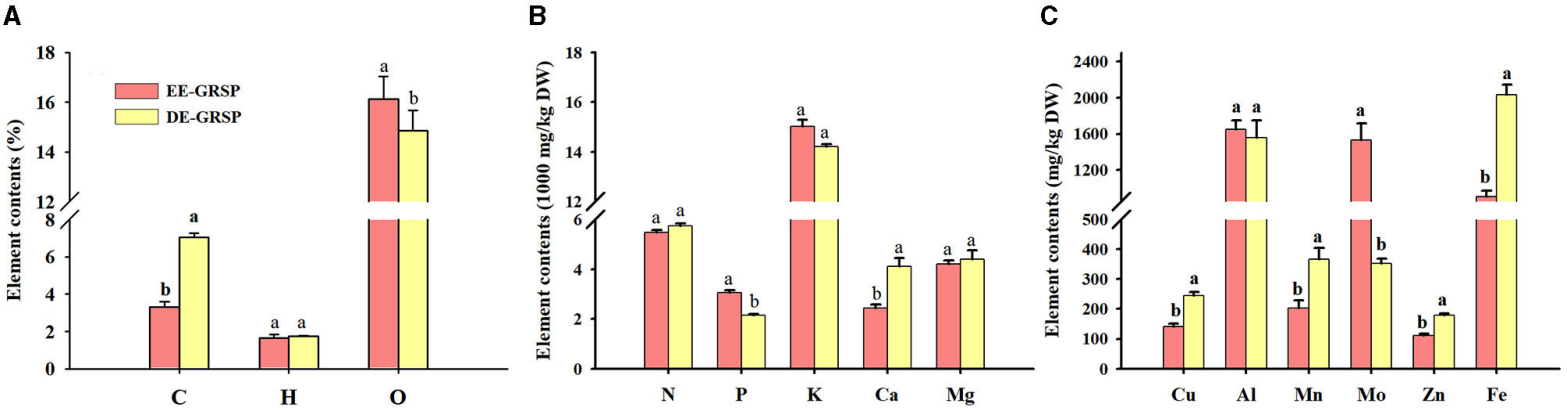
Differences in the element content [**(A)**: C, H, and O; **(B)**: N, P, K, Ca, and Mg; **(C)**: Cu, Al, Mn, Mo, Zn, and Fe] in purified EE-GRSP and DE-GRSP. Data (mean ± SD, *n* = 4) followed by different letters above the bars indicate significant differences (*p* < 0.05). DE-GRSP, difficultly extractable glomalin-related soil protein; EE-GRSP, easily extractable glomalin-related soil protein.

From the purified EE-GRSP and DE-GRSP, we collectively detected C, H, O, N, P, K, Ca, Mg, Cu, Al, Mn, Mo, Zn, and Fe, and some elements were below a detection concentration (Figures 5A–C). A significant difference in element content between purified EE-GRSP and purified DE-GRSP was observed. H, N, K, Al, and Mg content were not significantly different between two GRSP fractions. Purified DE-GRSP showed higher C, Ca, Cu, Mn, Zn, and Fe content than purified EE-GRSP by 52, 41, 43, 45, 38, and 55%, respectively. Purified EE-GRSP, however, had 9, 40, and 334% significantly higher O, P, and Mo content, respectively, than purified DE-GRSP.

### Changes in Root Auxins

**Figure 6:**
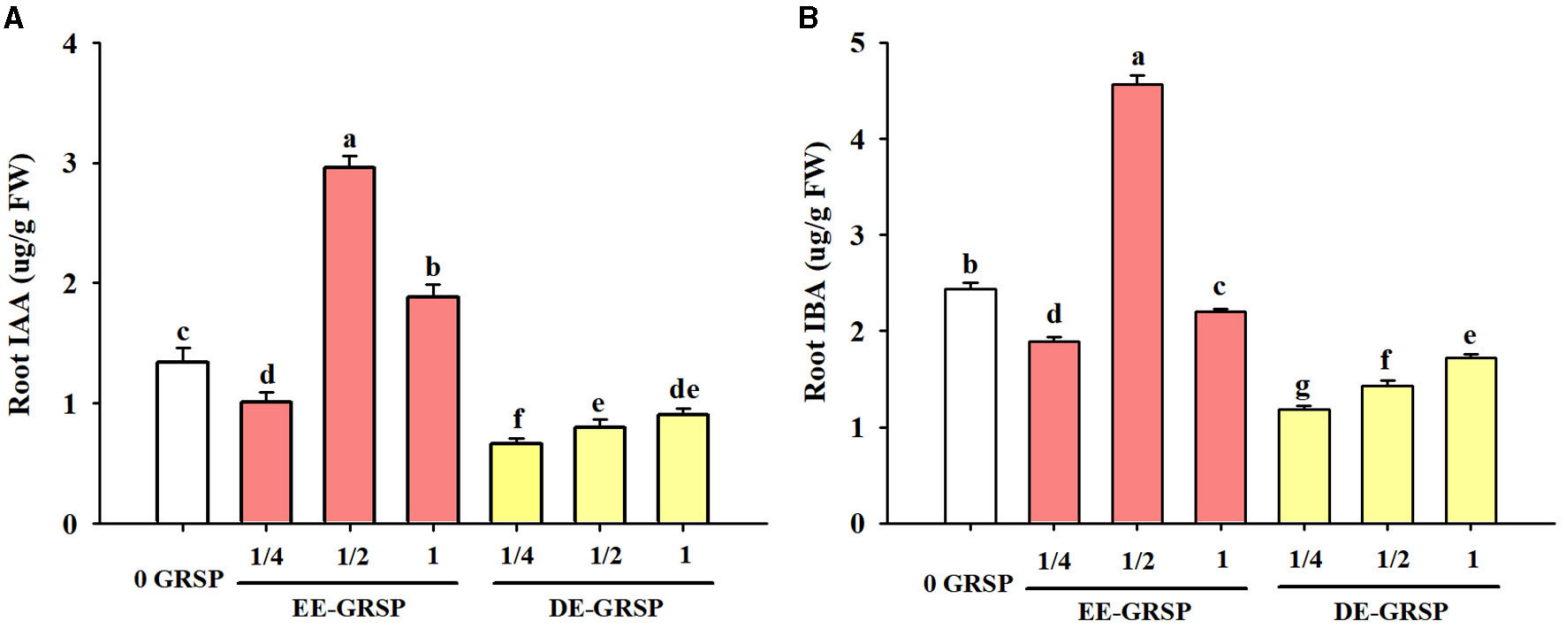
Effects of exogenous EE-GRSP and DE-GRSP on root IAA **(A)** and IBA **(B)** concentrations of trifoliate orange seedlings. Data (mean ± SD, *n* = 4) followed by different letters above the bars indicate significant differences (*p* < 0.05). DE-GRSP, difficultly extractable glomalin-related soil protein; EE-GRSP, easily extractable glomalin-related soil protein; IAA, indoleacetic acid; IBA, indolebutyric acid.

Compared with the control treatment, ¼ EE-GRSP treatment significantly reduced root IAA and IBA content by 25 and 23%, respectively (Figure 6); ½ EE-GRSP treatment registered an increase in root IAA and IBA content by 121 and 87%, respectively; and 1 EE-GRSP treatment recorded the increase in root IAA content by 40%, but decreased root IBA content by 10%. On the other hand, exogenous DE-GRSP application showed a decreasing trend on root IAA and IBA content. Compared with 0 GRSP treatment, ¼ DE-GRSP, ½ DE-GRSP, and 1 DE-GRSP treatment reduced root IAA content by 51, 40, and 33%, respectively, with the corresponding reduction in root IBA content by 52, 41, and 30%, respectively.

### Changes in Expression of Root Genes Associated With Auxin Synthesis

**Figure 7:**
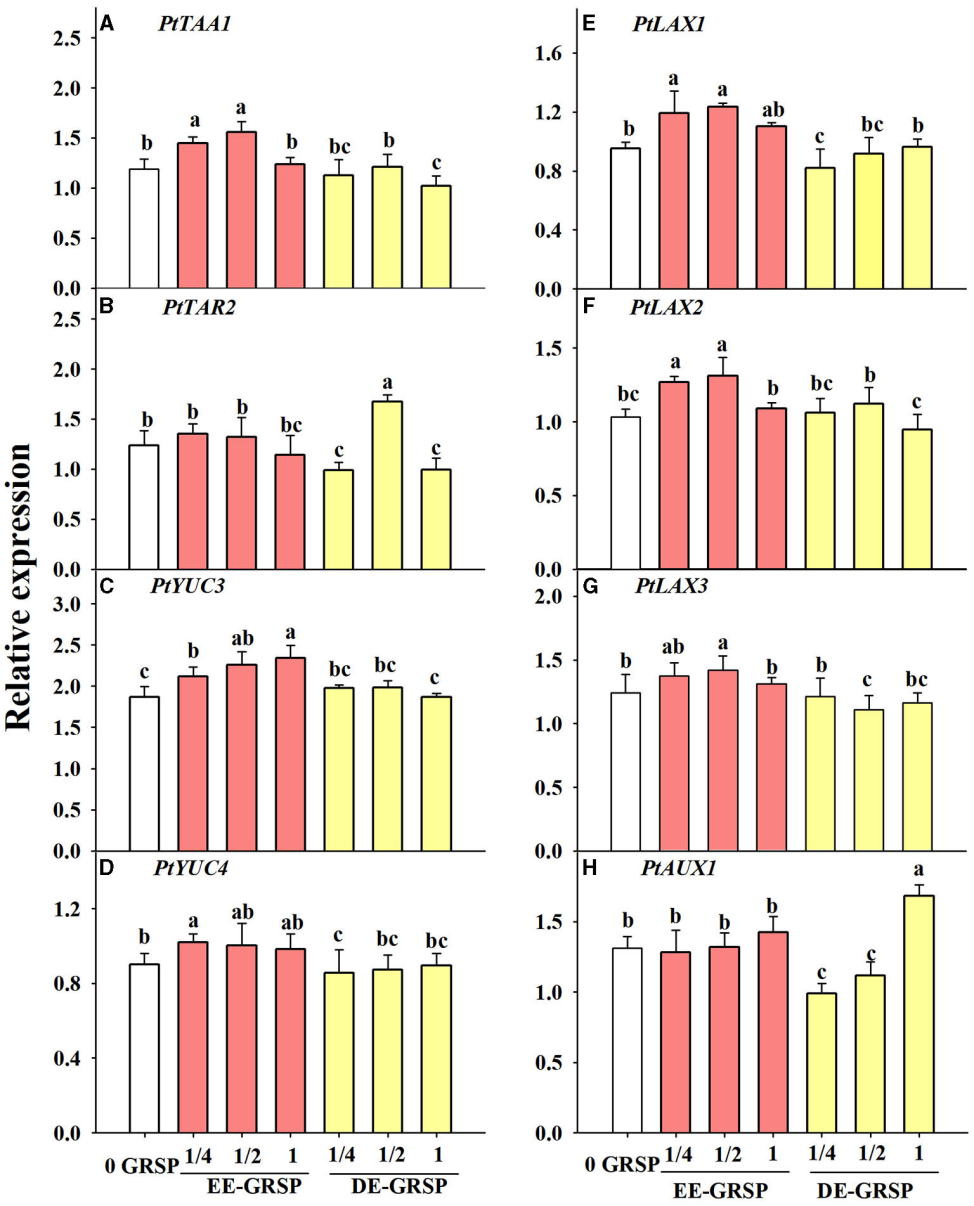
Effects of exogenous EE-GRSP and DE-GRSP on relative expression levels of auxin synthetase genes **(A–D)** and transporter protein genes **(E–H)** in roots of trifoliate orange seedlings. Data (mean ± SD, *n* = 4) followed by different letters above the bars indicate significant differences (*p* < 0.05). DE-GRSP, difficultly extractable glomalin-related soil protein; EE-GRSP, easily extractable glomalin-related soil protein.

Compared with the control, root *PtTAA1* expression was induced by exogenous ¼ EE-GRSP and ½ EE-GRSP, whereas it was inhibited by exogenous DE-GRSP with full strength (Figure 7A). Root *PtTAR2* expression was not affected by exogenous EE-GRSP, whereas it was downregulated by ¼ and 1 DE-GRSP application, accompanied by upregulated expression of *PtTAR2* by ½ DE-GRSP (Figure 7B). The *PtYUC3* expression was upregulated by exogenous EE-GRSP, along with increased expression with an increase in the concentration of EE-GRSP, but the *PtYUC3* expression remained unaffected by exogenous DE-GRSP, independent of its concentration (Figure 7C). The *PtYUC4* expression was increased by exogenous ¼ EE-GRSP but inhibited by ¼ DE-GRSP (Figure 7D).

### Changes in Expression Levels of Root Auxin Influx Transporter Protein Genes

Application of ¼ and ½ EE-GRSP treatment upregulated *PtLAX1* and *PtLAX2* expression, coupled with downregulated expression of *PtLAX1* with ¼ DE-GRSP and *PtLAX2* with 1 DE-GRSP, compared with the control (Figures 7E,F). Root *PtLAX3* expression was increased by ½ EE-GRSP while reduced by ½ DE-GRSP (Figure 7G). Root *PtAUX1* expression was induced by 1 DE-GRSP, whereas inhibited by ¼ and ½ DE-GRSP (Figure 7H).

### Correlation Between Root IAA Concentration and Gene Expression Levels

Root IAA was significantly and positively correlation with *PtTAA1* (*p* < 0.01), *PtYUC3* (*p* < 0.01), *PtYUC4* (*p* < 0.05), *PtLAX1* (*p* < 0.01), and *PtLAX3* (*p* < 0.01), respectively (Table 4). However, there was no any significant correlation of root IAA with *PtTAR2, PtLAX2*, and *PtAUX1*.

**Table 4:** The Pearson's correlation coefficient between root IAA and expressions of auxin synthetase genes and carrier genes (*n* = 28).

	**Genes associated with auxin synthesis**				**Auxin influx carrier genes**			
	***PtTAA1***	***PtTAR2***	***PtYUC3***	***PtYUC4***	***PtLAX1***	***PtLAX2***	***PtLAX3***	***PtAUX1***
Root IAA	0.62^**^	0.07	0.61^**^	0.40^*^	0.63^**^	0.47	0.53^**^	0.21

*IAA, indoleacetic acid*.

* *p < 0.05;*

** *p < 0.01*.

## Discussion

In this study, we firstly observed a difference in plant growth response of trifoliate orange with exogenous application of two GRSP fractions. Exogenous EE-GRSP strongly promoted plant growth, whereas exogenous DE-GRSP distinctly inhibited plant growth. Such results provide important clues in favor of EE-GRSP functioning as a growth promoter of trifoliate orange. The increase in plant growth response to EE-GRSP treatments was consistent with the findings of Wang et al. (2015). It is well-known that GRSP contained an amount of humic acid (Gadkar and Rillig, 2006), similar to NMR spectrum-based humic acid (Schindler et al., 2007). Humic acid as an important component of humic substance, a part of natural organic matter, could improve the growth performance of various plants (Spohn and Giani, 2010; Mora et al., 2012; Xu et al., 2012; Duan, 2014; Dong et al., 2016). It is believed that EE-GRSP having a parallel NMR spectrum-based humic acid-like substance, functions in increasing plant growth. On the other hand, plant height, stem diameter, leaf number, and biomass production decreased significantly after the application of DE-GRSP, and the inhibitory effect increased with the increase of exogenous DE-GRSP concentration. Perhaps DE-GRSP contains some impurities (Gillespie et al., 2011) that are detrimental to plant growth, but it is not clear about the nature of those impurities responsible for such undesirable plant responses. EE-GRSP, an active form of newly produced glomalin, and DE-GRSP, a recalcitrant and older glomalin (Wu et al., 2015a), also contribute primarily toward difference in magnitude of plant response. More studies have to be conducted to analyze the difference in molecular structure and relevant properties between EE-GRSP and DE-GRSP.

In the present study, leaf total chlorophyll content was differentially affected by EE-GRSP and DE-GRSP. Exogenous EE-GRSP significantly increased total chlorophyll content, and the most significant effect was observed with ½ EE-GRSP. In contrast, exogenous DE-GRSP caused a significant decrease in total chlorophyll content. Our study also revealed that GRSP contained many mineral elements, such as Fe and Mg, which are part of chlorophyll (Schindler et al., 2007). In addition, EE-GRSP as a heat-shock protein-like substance could keep the stability of PSII complexes and thylakoid membrane, which plays an important role in chlorophyll functioning. Chi et al. (2018) proposed that EE-GRSP as a molecular chaperone improved plant photosynthetic efficiency. On the contrary, DE-GRSP is an inert substance recalcitrant in the soil, which possibly induced a disadvantageous influence on chlorophyll production. The content of Fe in purified DE-GRSP was significantly higher than that in purified EE-GRSP. Nevertheless, it still showed that exogenous DE-GRSP inhibited the chlorophyll content, hinting toward another unknown mechanism to be perused as future research.

We investigated the elemental composition alongside their concentrations of purified GRSP, revealing the presence of as many 14 elements, of which P, K, Mo, and O were higher in purified EE-GRSP than in purified DE-GRSP. Other nutrients, viz., C, Ca, Cu, Mn, Zn, and Fe, displayed higher concentrations in purified DE-GRSP than in purified EE-GRSP. The variation in element contents of two GRSP fractions constituted different organic substances (e.g., tyrosine, tryptophan, fulvic acid, humic acid, nitrobenzoxadiazole, and calcofluor) (Zhang et al., 2017b), thus presenting such contrasting growth responses.

The root nutrient acquisition of trifoliate orange was differentially regulated by exogenous GRSP: an increase in N, P, K, Ca, and Fe under EE-GRSP application conditions and a reduction in N, P, K, Mg, Cu, Zn, and Fe under DE-GRSP application conditions. As reported by Chi et al. (2018), ½ EE-GRSP improved the morphological establishment of trifoliate orange roots under normal water and drought stress. Exogenous EE-GRSP also improved soil aggregate stability and soil enzyme activity (Wang et al., 2015), both of which are beneficial to plant growth and nutrient acquisition. Root Fe was elevated by exogenous EE-GRSP, amounting to 1.8–4.9%, and inhibited by exogenous DE-GRSP, amounting to 59.1–77.9%. However, purified DE-GRSP possessed 55% higher Fe than purified EE-GRSP, gluing more Fe into an organic state to render Fe into inaccessible form to be absorbed by plant roots. Our study also showed that EE-GRSP-applied plants maintained a relatively higher root total length, surface area, and volume than the control plants, with ½ EE-GRSP presenting the best effect. However, DE-GRSP dramatically inhibited the root morphology, and the root morphology declined with the increasing concentration of DE-GRSP. The changed trend of root morphology was surprisingly consistent with the plant growth performance and chlorophyll changes triggered by GRSP application. As a result, it was concluded that changes in plant growth response and root nutrient acquisition under GRSP application are associated with root morphology triggered after GRSP application.

Plant growth response is closely linked to endogenous auxins (Zheng et al., 2013; Chen et al., 2016). In the present study, exogenous ½ and 1 EE-GRSP significantly increased root IAA and IBA contents, due to the presence of tryptophan (the precursor of auxins) in purified EE-GRSP (Zhang et al., 2017b), accelerating the synthesis of auxins. Chi et al. (2018) also observed an increase of IAA in trifoliate orange under soil water deficit conditions, following the application of exogenous EE-GRSP. However, exogenous application of DE-GRSP deteriorated the soil environment for roots to absorb nutrients (Zhang, 2009), thus hampering the bidirectional transport of IAA in the phloem.

The synthesis of IAA in plants starts with the conversion of L-tryptophan into 3-indole pyruvic acid (IPyA) in the presence of tryptophan transcarbamylase (tryptophan aminotransferase related, TAA1/TAR), which is then converted into IAA under the influence of flavin monooxygenase-like enzymes (YUCs) (Brumos et al., 2014). In the present study, exogenous application of EE-GRSP induced root *PtTAA1, PtYUC3*, and *PtYUC4* expression, depending upon the concentration of EE-GRSP used, whereas exogenous DE-GRSP inhibited the relative expression of *PtTAA1* and *PtYUC4*. The GRSP-regulated IAA change was related to the expression of GRSP-induced auxin-related synthase genes (*PtTAA1, PtYUC3*, and *PtYUC4*), based on the correlation analysis. TAA is involved in the conversion of tryptophan into IpyA, and then YUCs convert IpyA into IAA (Brumos et al., 2014). It indicates that the two processes were regulated by exogenous GRSP. Indeed, the IAA synthesis process is regulated by various transcription factors, such as the phytochrome-interacting factor 4 (*PIF4*) for direct regulation of *TAA1* gene expression and the *PIF7* for regulation of *YUCs* (Li et al., 2012; Sun et al., 2012), implying that exogenous GRSP regulated *PIF* transcription factors to modulate the expression of *TAA* and *YUCs*. However, the evidence of direct involvement is lacking in our study, and further research is needed in this direction.

In plants, IAA enters into cells in the form of anion under the influence of endocytosis carrier genes (Delbarre et al., 1996). *Auxin residue 1*/*like AUX1* (*Aux*/*LAX*) genes play an important role in auxin influx (Yoshihiro and Keiichirou, 2012). The results of our study showed that the application of exogenous EE-GRSP with quarter and half strength upregulated the expression of *PtLAX1* and *PtLAX2* and did not affect the expression of *PtAUX1*, suggesting *LAX* genes could be induced by EE-GRSP. However, exogenous DE-GRSP, to some extent, decreased the expression of *PtLAX* genes, dependent on the combination of DE-GRSP concentration and gene species, likely to reduce the flow of IAA from shoot parts to roots. A positive correlation of root IAA with *PtLAX1* and *PtLAX3* suggested the stimulating effect of GRSP on the expression of IAA influx carrier genes. The *AtLAX1* in *Arabidopsis thaliana* was expressed in the vascular system of the primary root maturation zone but weakly expressed at the root tip (Peret et al., 2012). The expression of *AtLAX3* at the cortex of the newly lateral root primordia mediated auxin transport during lateral root primordia development, making the formation and development of lateral roots easier (Swarup et al., 2008). Higher expression of *PtLAX1* and *PtLAX3* in EE-GRSP-treated plants indicated better polar auxin transport and lateral root formation, as seen in greater root morphology in EE-GRSP-treated plants. Such results also showed that the change of root IAA in response to the GRSP application is associated with regulation of IAA in polar transport from shoots to roots, besides aiding in the formation of lateral roots.

## Conclusion

Exogenous GRSP triggered differential effects on plant growth response of trifoliate orange: the increase by exogenous EE-GRSP and the reduction by exogenous DE-GRSP. The change in plant growth under GRSP application conditions was associated with the element content of pure GRSP, the auxin content of the plant, and root morphology. GRSP-regulated IAA changes were associated with the GRSP regulation of *TAA* and *YUC* gene expression in the IAA synthesis pathway and polarity transport of IAA. These results provide the future possibility of using EE-GRSP as a plant growth promoter for citrus production. However, more field studies coupled with elaboration on the physiological basis of GRSP functioning and mechanism of growth response by GRSP are mandatory before such efforts become an accepted field practice as a technological novelty.

## Data Availability Statement

The original contributions presented in the study are included in the article/supplementary material, further inquiries can be directed to the corresponding author.

## Author Contributions

R-CL, W-QG, and Q-SW designed the experiment. W-QG prepared the materials for the experiment. R-CL, Y-NZ, KK, and Q-SW analyzed the data. R-CL wrote the manuscript. AS, AH, EA, Q-SW, and KK revised the manuscript. All authors have read and agreed to the published version of the manuscript.

## Funding

This study was supported by the National Natural Science Foundation of China (31372017) and the 2020 Joint Projects between Chinese and CEECs' Universities (202019). This work was also supported by the UHK project VT2019-2021. The authors would like to extend their sincere appreciation to the Researchers Supporting Project Number (RSP-2021/134), King Saud University, Riyadh, Saudi Arabia.

## Conflict of Interest

The authors declare that the research was conducted in the absence of any commercial or financial relationships that could be construed as a potential conflict of interest.

## Publisher's Note

All claims expressed in this article are solely those of the authors and do not necessarily represent those of their affiliated organizations, or those of the publisher, the editors and the reviewers. Any product that may be evaluated in this article, or claim that may be made by its manufacturer, is not guaranteed or endorsed by the publisher.
